# Does robotic technology successfully restore the joint line after total knee arthroplasty? A retrospective analysis

**DOI:** 10.1186/s42836-021-00103-6

**Published:** 2022-01-05

**Authors:** Varun O. Agrawal, Anup P. Gadekar, Narendra Vaidya

**Affiliations:** 1 Joint Replacement and Trauma Department, Lokmanya Hospital, 484/6, Shiv Darshan Rd, Mitra Mandal Colony, Parvati Paytha, Pune, Maharashtra 411009 India; 2 Department of Arthroplasty, Lokmanya Hospital, 484/6, Shiv Darshan Rd, Mitra Mandal Colony, Parvati Paytha, Pune, Maharashtra 411009 India

**Keywords:** Robotic-assisted knee arthroplasty, Robotic technology, Joint line restoration, TKR, TKA, Total knee replacement

## Abstract

**Background:**

Our study aims to determine the effectiveness of robotic technology for total knee arthroplasty in the successful restoration of the joint line of the knee with respect to that of a normal human anatomical knee. The restoration of the joint line is an important technical goal on which the postoperative outcomes and the success of the surgery depend.

**Methods:**

Sixty-four postoperative plain anteroposterior radiographs of 60 patients, who received total knee arthroplasty by using the robotic technology were analyzed and compared with 66 similar radiographs of 60 patients who received the conventional method. The distances of the lateral epicondyle to the joint line (LEJL) and proximal tibiofibular joint to the joint line (PTFJJL) were calculated and analyzed.

**Results:**

We found that the mean value of LEJL minus PTFJJL in the robotic group was 0.334 ± 0.115 (mean ± SD), while in the conventional group, it was 2.304 ± 0.308. The difference between the two groups was statistically significant. The mean ratio (LEJL:PTFJJL) in the robotic group was also equal to 1.017 ± 0.042.

**Conclusion:**

From these findings it could be concluded that the robotic technology significantly increases the accuracy of the total knee arthroplasty and, compared to the conventional method, achieves an almost anatomical position of the joint line.

## Background

Restoring joint line is an important goal of total knee arthroplasty (TKA) [1]. Failure to restore the joint line to its anatomical position can result in anterior knee pain, mid-flexion instability, reduced range of motion, and patellar mal-tracking [1]. All of this leads to lower knee scores and lower patient satisfaction. The success of a total knee arthroplasty depends upon the restoration of the normal knee kinematics. Various modifications to conventional methods, implant designs, and instruments are in an ongoing process to achieve the same goals. Many computer-assisted and navigational devices have been introduced to date. One of such advances is the introduction of robotic technology. This technology serves as an intraoperative guidance tool for the operating surgeon and allows foran objective assessment at every step of the procedure, thus, increasing the precision of the surgery [2]. Not many studies have been carried out to analyze these robotic systems with regard to their effectiveness and their contribution to the success of the surgery. The aim of this retrospective study was to analyze how precisely the joint line is restored using robotic technology for the primary total knee replacement.

## Methods

Plain anteroposterior (AP) radiographs of 64 knees from 60 patients who had undergone total knee replacement using robotic technology between November 2017 and March 2020 were analyzed and compared with the AP radiographs of 66 knees of 60 patients who had undergone a TKA using the conventional method during the same period. The consent of all the patients was obtained for the study.

Inclusion criteria: (1) Cases who received total knee arthroplasty by using robotic technology  or conventional methods; (2) Those who were preoperatively diagnosed as having primary knee osteoarthritis with genu varus deformity and fixed flexion deformity of less than 15°; (3) Patients whose true postoperative AP radiographs were available.

Exclusion criteria: (1) Subjects who were preoperatively diagnosed with post-traumatic osteoarthritis and inflammatory arthritis; (2) Those who had preoperative valgus knee deformity; (3) Candidates who had pre-existing hip pathology and hip arthroplasty.

The operative procedure, using robotic technology, begins with connecting the hardware, selecting bur and control, and calibrating the handpiece. Surgical choice was according to surgeon's  preferences. We used the anterior referencing system, the posterior condylar axis as the reference axis for rotation and the resection depth equal to the implant thickness. The exposure was achieved by using the medial parapatellar approach and patelloplasty was done. Trackers were attached and bony landmarks were marked for the femur and tibia. The hip center was calculated. The preoperative range of motion of the knee was recorded. Afterwards, the femoral and tibial articular surfaces were then mapped. Virtual images were then used to plan on the screen. We aimed at achieving < 1–2 mm of joint laxity in the lateral compartment over the entire range of motion of the knee. Femoral and tibial cuts were then made with a 5 mm bur according to the final plan. Trials were then taken and the sizes were confirmed. Cementation and final implantation were  performed. The robotic system (Smith & Nephew Navio PFS, Blue Belt Technologies, Plymouth, MN)is a haptic system. The implants used were Anthem or Genesis II, CR (cruciate retaining)/PS (posterior-cruciate sacrificing) knee implants.

The proximal tibiofibular joint (PTFJ) and the lateral epicondyle (LE) of the femur were used as anatomical reference points for the analysis of the joint lines on the X-ray images. The PTFJ can be identified as the center of the horizontal portion of the proximal tibiofibular joint [3]. If it is not visible due to fibular rotation, it is at the intersection of the lateral prominence of the fibular head and the fibular styloid [3, 4]. LE is the most prominent bony point on the lateral distal femur, from which the lateral collateral ligament originates [3, 4]. The joint line (JL) is represented in the AP X-ray image by a line drawn tangentially to the distal surface of the femoral prosthesis. The distances of the joint line from the lateral epicondyle of the femur (LEJL) and the proximal tibiofibular joint (PTFJJL) were measured and compared. If the joint line is at the same distance from these two bony landmarks, it can be said that the joint line was successfully restored [4]. All data were calculated and collected by the corresponding author only. SCANDOC DICOM 5.2.0.0 and MICRODICOM 3.1.4 software packages were used as calculation software.

We analyzed the postoperative functional outcome of the surgery in both the robotic and conventional groups using the Oxford Knee Score (OKS), which consists of 12 questions [5–7]. The score rates the pain status and activity level of the knee, as reported by the patient. Each question was rated between 0 (the worst outcome) and 4 (the best outcome). All scores were summed up to give the final score which ranged between 0 (the worst outcome) and 48 (the best outcome) [5–7].

The probability value < 0.05 was considered as a significant level for all the statistical analyses. The results were analyzed using an unpaired *t*-test and ANOVA. The level of evidence of our study is 3.

## Results

**Fig. 1:**
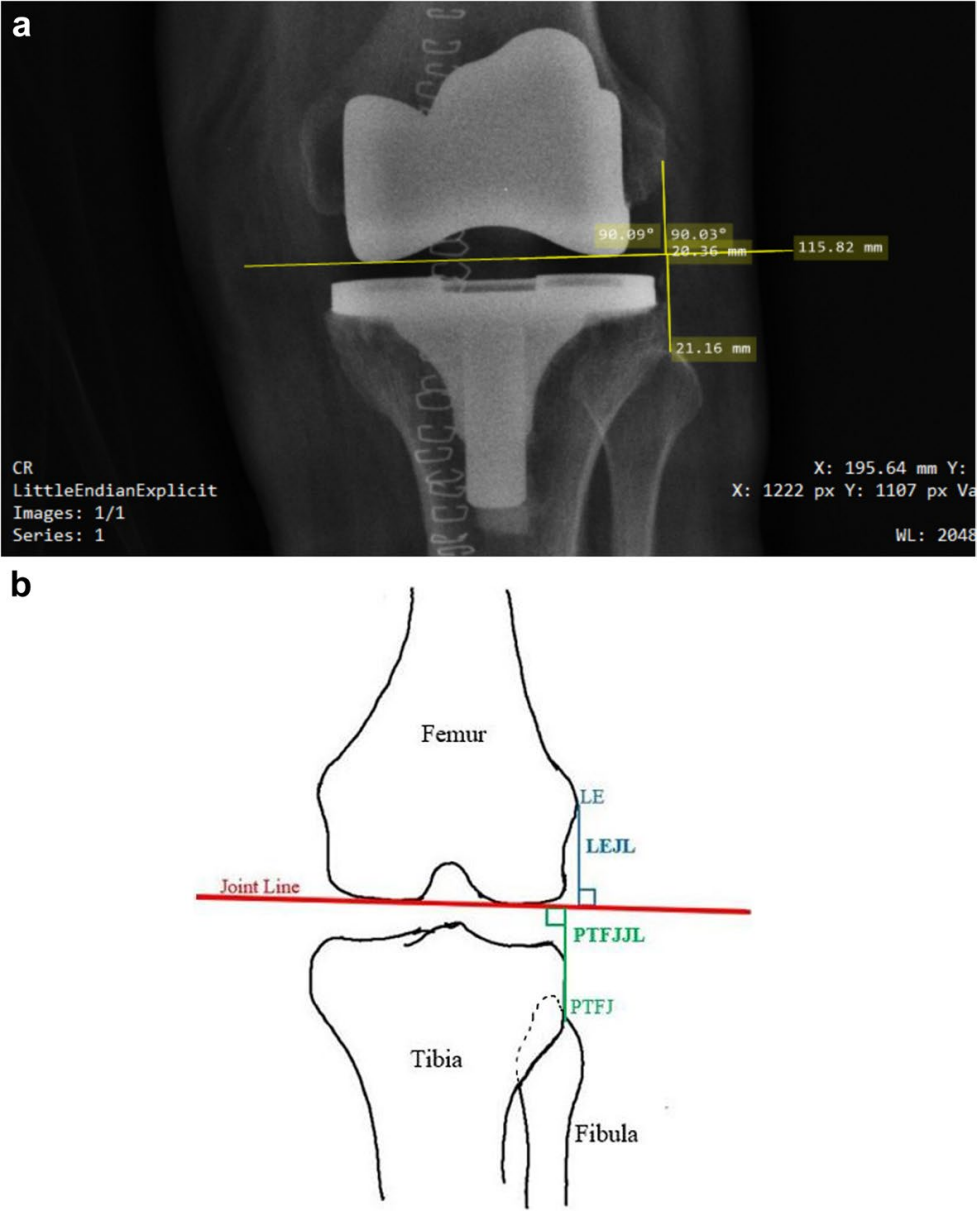
**a** Plain AP radiograph of a patient’s left knee from the sample showing how the LEJL and PTFJJL distances were calculated. The horizontal line is the joint line achieved after the surgery. The perpendicular above this line is LEJL and the perpendicular below this line is PTFJJL. Both these distances are nearly equal*. ***b** Sketch diagram of a left knee showing how the LEJL and PTFJJL distances were calculated. The horizontal line is the joint line (red) of the knee. The perpendicular above this line is LEJL (blue) and the perpendicular below this line is PTFJJL (green). Both these distances are nearly equal

There was no significant difference in the demographic data between the patients operated with the robotic technology (R-TKA) and the conventional method (C-TKA) (Table 1). Forty-four of 64 joints (68.75%) in the R-TKA group had absolute values of LEJL minus PTFJJL < 1 mm. The value of LEJL minus PTFJJL in this group was 0.334 ± 0.115 (mean ± SD) (Table 2). Using the unpaired *t*-test, this difference was found to be insignificant (*P* = 0.41). The value of (LEJL:PTFJJL) in the robotic group was 1.017±  0.042. Thus, with both methods, we found that both the distances are nearly equal, which suggests that the joint line lies equidistant from both these two points, which confirms the accuracy of the robotic method. The value of LEJL minus PTFJJL in the conventional group was 2.304 ± 0.308. The difference between the mean values in the robotic and conventional groups turned out to be highly statistically significant (*P* < 0.001) (Table 2). We also found that the precision of this method was independent of the age, gender, body mass index (BMI), and side of the operated patient (Tables 3, 4, 5, 6). We divided the data into different comparison groups within these demographic categories and analyzed the means of LEJL minus PTFJJL in these groups. We found that the difference was not significant. Figure 1a and b show the method used for calculating the LEJL and PTFJJL distances.

**Table 1:** Comparison of demographic characteristics between the R-TKA and C-TKA groups (N.S. = not significant)

Demographic parameters	R-TKA	C-TKA	*P* value
Age (mean ± SD)	68.97 ± 8.52	68.37 ± 8.48	0.69 (N.S.)
BMI (mean ± SD)	26.94 ± 2.65	26.56 ± 2.76	0.44 (N.S.)
Gender (M:F)	22:38	20:40	0.70 (N.S.)
Side (Right: left)	33:31	26:40	0.16 (N.S.)

**Table 2:** Comparison of the mean values of LEJL minus PTFJJL between the patients receiving robotic technology and those receiving the conventional method (H.S. = highly significant)

Technique	*n*	Mean	SD	*P* value (Unpaired *t*-test)
R-TKA	64	0.334	0.115	< 0.001 (H.S.)
C-TKA	66	2.304	0.308

**Table 3:** Comparison between the mean values of LEJL minus PTFJJL in different age groups of the R-TKA group

Age group	*n* (%)	Mean	SD	*P* value (One way ANOVA)
< 60	9(14%)	0.884	0.739	0.45 (N.S.)
60–75	42(66%)	0.112	0.963	
> 75	13(20%)	0.671	0.641

**Table 4:** Comparison between the mean values of LEJL minus PTFJJL with respect to BMI in the R-TKA group

BMI	*n* (%)	Mean	SD	*P* value (One way ANOVA)
18.5–24.9	12(19%)	0.485	0.725	0.061 (N.S.)
25–29.9	43(67%)	0.167	0.962	
> 30	9(14%)	0.933	0.753

**Table 5:** Comparison of mean values of LEJL minus PTFJJL between male and female genders in the R-TKA group

Sex	*n* (%)	Mean	SD	*P* value (Unpaired *t*-test)
Male	22(34%)	0.559	0.247	0.16 (N.S.)
Female	42(66%)	0.167	0.19

**Table 6:** Comparison of mean values of LEJL minus PTFJJL with respect to right or left knee operated in the R-TKA group

Side	*n* (%)	Mean	SD	*P* value (Unpaired *t*-test)
Left	31(48%)	0.535	0.822	0.092 (N.S.)
Right	33(52%)	0.146	0.984

In the postoperative analysis with the OKS scoring system, we found that 12 months after operation,  in the R-TKA group, 68.25% of the knee scores were between 40 and 48 and 31.75% of the scores were between 30 and 39. While in the C-TKA group, 51.61% of the knee scores were between 40 and 48 and 48.39% of the scores were between 30 and 39. The mean OKS value in the R-TKA group was found to be 40.87 ± 4.675, while in the C-TKA group, the mean OKS value was found to be 37.65 ± 5.125. We lost 3 cases to follow-up in the R-TKA group and 4 cases in the C-TKA group.

## Discussion

The results of our study are broadly in line with what was reported by Gavin *et al* [3] According to their findings, the joint line of a normal knee is equidistant from the LE and PTFJ with a mean of 1.0 ± 0.1 for the ratio of LEJL:PTFJJL. Figgie *et al* [8] had first suggested the importance of restoring joint line in their study and found that a variation in joint line of more than 8 mm can lead to poorer results in primary total knee arthroplasty (TKA). Similarly, in their 1999 study, Partington *et al* [9] suggested  < 8 mm as an acceptable variation in the joint line for better results. While Hofmann *et al* [10] suggested in 2006 that the elevation or depression of the joint line should not be more than 4 mm. In our study, we took the joint line in a normal human anatomical knee as described by Gavin *et* *al* [3] as a reference, and found that the maximum variation in the joint line after robotic-assisted primary TKA is < 2.5 mm compared to the reference, with 68.75% of the cases showing a deviation of < 1 mm and 26.56% of the cases showing a deviation of 1–2 mm. Three cases (4.68%) showed variations between 2 and 2.5 mm.

The success of TKA depends upon soft tissue balancing, bone cuts, cementation, and restoration of the joint line. The knee joint is a hinge and pivot type of joint with 6 degrees of freedom: 3 rotations and 3 translations. Any change in the joint line and angle can lead to joint instability and failure of the surgery [1, 11]. As shown in previous studies, a joint line variation of more than 8 mm [8, 9, 12] or more than 4 mm [10] leads to poorer results. Chiu *et al* reported that elevation of the joint line by more than 10 mm leads to a reduction in the flexion range by more than 25% [1, 13]. Restoring the joint line to the near-normal results in improved knee scores [9]. Amarnath *et al* [4] mentioned that conventional methods lack accuracy and lead to the mal-positioned and frequently elevated joint line. Elevation of the joint line leads to many problems such as anterior knee pain, reduced range of motion (ROM), patella baja, mid-flexion instability [14], patellar tendon impingement, and accelerated wear [1]. Hence, it is important to restore the joint line near normal for better outcomes of the procedure.

Several methods have been described for analyzing the joint line [4]. Earlier studies attempted to analyze the joint line,  taking into account various bone and soft tissue landmarks and ratios [3, 4, 15–18]. Rajagopal *et al* [16] and Mountney *et al* [17] suggested that the ratio between inter-epicondylar distance (IED) and perpendicular to the joint line from the medial epicondyle or lateral epicondyle is constant and is about 3.0. However, Servien *et al* [18] found that this distance was variable depending upon the size and gender of the individual. He also found that there is a wide variation in the bony landmarks. The medial epicondyle is a sulcus between two protuberances and thus an inconsistent landmark [3, 4]. LEJL is therefore regarded as the most reliable distance for assessing the restoration of the joint line [3, 4]. There is no consensus on the exact reference point on the fibular head [19, 20]. Also, the fibular styloid has a variable morphology [3, 4, 18]. Therefore, Amarnath *et al* [4] and Galvin *et al* [3] suggested, from their studies, that the most effective method of analyzing a successful restoration of the joint line is to determine the distance of the joint line from LE and PTF joint. If these two distances are nearly equal, it confirms the successful restoration of the joint line [3, 4]. We used this method to analyze the joint line.

The robotic system enables the surgeon to assess the knee kinematics, in all 6-degrees of freedom of movement on the screen, for instance, the ROM, mediolateral laxity, and alignment of the joint over the entire ROM and also the three-dimensional view of the condyles of the femur and tibia by collecting the femoral and tibial collection points. Depending on the surgeon’s preference, he or she can customize the settings for cruciate ligament-retaining or cruciate ligament-substituting methods before the start of the procedure. The system gives guidance about the further steps of the procedure accordingly. The complete planning of the cuts to be made, the position of the implants, the size of the components, the resulting limb alignment, mediolateral laxity, and the ROM of the knee, that will be achieved at the end of the implantation, can be calculated on the planning screen before any cuts are made. Any femoral notching, component overhang, or malrotation is easily noticeable on the screen. The cutting zigs are also placed under the guidance of the system. The use of the system thus avoids the excessive soft tissue dissection required to meet the surgeon’s need for exposure and also avoids the over- or under-resection of the bone to compensate for the inadequate soft tissue releases and *vice* *versa*, as seen in the conventional method in which all these measurements are taken manually by the surgeon using the available guides from the implant system. The final results achieved after the implant has been positioned can also be analyzed on the robotic system in order to rule out errors during the implantation. This technology therefore combines both measured resection and gap balancing techniques and the surgeon does not have to rely on a single technique. In the conventional method, the analysis is dependent more on the surgeon’s subjective findings and therefore increases the likelihood of interpersonal errors. The accuracy of a surgeon’s analysis in the conventional method increases with his experience and knowledge. In the case of robotic technology, however, once a surgeon is familiar with the system, the analysis is objective, as shown on the screen, and its accuracy does not vary with the surgeon’s experience and therefore does not affect patient outcomes.

Recent studies also showed that the use of robotic technology does not lead to increased surgical time or complications [21–25]. Coon *et al* have come to the conclusion that once the surgeon gets familiar with the robotic technology, the operative time is reduced to < 40 min [26]. Robotic technology is intended to assist the operating surgeon and not to replace him [27]. The use of robots in the medical field dates back to the late 1980s [28]. Robodoc (Curexo Technology, Fremont, CA) was the first robot to be used in total hip and knee arthroplasty in 1992 [21]. However, the versatility of this system was less, which, along with increased operative time and complexity of the system, could not make it very popular amongst the surgeons [21, 29]. To overcome these shortcomings, many new robotic systems have been developed that allow for greater precision in femoral and tibial cuts. Navio PFS (Blue Belt Technologies, Plymouth, MN) is one of such robotic systems. In contrast to navigation systems, robotic systems use programmable devices that provide the surgeon with continuous feedback on the spatial position and orientation of the instruments, guiding the procedure, and following the surgical plan. This helps in achieving better kinematic alignment, mechanical alignment [24, 30, 31], and soft tissue balancing [2, 21, 28, 30]. This, in turn, helps to achieve better implant survival, better knee function, and better patient-reported outcomes [22].

Robotic-assisted knee replacement technology helps reduce postoperative pain [24, 30–33], restore knee function, and thus improve the quality of life in patients with severe osteoarthritis [34]. Khlopas *et al* [35] and Kayani* et al* [25] have suggested, in their studies, that these systems also reduce excessive soft tissue trauma during surgery compared to the conventional systems and thus reduce the postoperative pain. According to a retrospective study involving 3100 navigated TKA, the prevalence of fractures at the pin-insertion site is 0.16% and infection at the pin-site is 0.47% [36]. However, the data on the prevalence of these complications in robotic systems are still insufficient. To date, robotic-assisted TKA has not been shown to be cost-effective due to several factors involved [37]. However, it can become cost-effective when used in high volume centers where the major capital is recovered in the first year [22, 37, 38] and if it averts the risk of revision surgery [21, 39]. The effective benefits of this technology are still being explored and, once proven, can help determine the risk of revision surgery following robotic-assisted knee replacement surgery.

Despite the above results, our study still have a few limitations. First, in this study we could not compare the postoperative Oxford Knee Score to the preoperative score because the preoperative scoring data were not available as the study was retrospective. Secondly, there was a deviation of 0.01–0.1 degrees in the perpendiculars drawn from the LE and PTFJ to the joint line (software errors). However, this variation was very small and did not influence the overall observations.

## Conclusion

We were thus able to deduce from our study that the use of robotic technology in total knee replacement surgery achieves a nearly anatomical position of the joint line and the accuracy of the surgery can be significantly increased compared to the conventional method. The effects on patient-related outcomes and the need for revision surgery compared to the conventional method need to be further analyzed.

## Data Availability

The data sets used and / or analyzed in the current study are available from the corresponding author on reasonable request.

## Abbreviations

LEJL: Lateral epicondyle to the joint line
PTFJJL: Proximal tibiofibular joint to the joint line
TKA: Total knee anthroplasty
AP: Anteroposterior
CR: Cruciate-retaining
PS: Posterior-cruciate sacrificing
PTFJ: Proximal tibiofibular joint
LE: Lateral epicondyle
JL: Joint line
R-TKA: Robotic total knee anthroplasty
C-TKA: Conventional total knee anthroplasty
BMI: Body mass index
ROM: Range of motion
IED: Inter-epicondylar distance
OKS: Oxford Knee Score
